# Effects of the PA-X and PB1-F2 Proteins on the Virulence of the 2009 Pandemic H1N1 Influenza A Virus in Mice

**DOI:** 10.3389/fcimb.2019.00315

**Published:** 2019-09-03

**Authors:** Jun Ma, Shun Li, Kangjian Li, Xiangbin Wang, Shoujun Li

**Affiliations:** ^1^College of Veterinary Medicine, South China Agricultural University, Guangzhou, China; ^2^Guangdong Provincial Key Laboratory of Prevention and Control for Severe Clinical Animal Diseases, Guangzhou, China; ^3^Guangdong Technological Engineering Research Center for Pet, Guangzhou, China; ^4^School of Life Science and Engineering, Foshan University, Foshan, China

**Keywords:** influenza, PA-X, PB1-F2, mice, host-shutoff

## Abstract

There have been several previous reports showing that PA-X and PB1-F2 proteins can regulate innate immune responses and may play roles in the adaptation of influenza viruses to new hosts. In this research, we investigated, for the first time, the combined effects of PA-X and PB1-F2 proteins on viral virulence in mice. Based on the 2009 pH1N1 A/Guangdong/1057/2010 virus backbone, four viruses encoding different combinations of full-length or truncated PA-X and PB1-F2 proteins were rescued by a reverse genetic engineering system. We analyzed viral replication, host-shutoff activity, *in vitro* viral pathogenicity and *in vivo* host immune response. We found that simultaneously expressing the full-length PA-X and PB1-F2 proteins enhanced viral replication *in vitro* through increasing the accumulation of the RNP complex protein and enhanced viral pathogenicity in mice during the early stage of infection. Furthermore, PA-X and PB1-F2 simultaneously regulated the host innate response, and different forms of PB1-F2 proteins may have impacts on the host shutoff activity induced by the PA-X protein. Our results provide a better understanding of the mechanisms of PA-X and PB1-F2 proteins during viral replication, pathogenicity and host immune response.

## Introduction

Influenza A virus circulates in multiple hosts ranging from aquatic or terricolous birds to diverse mammalian species, including humans. Influenza A virus contains eight single negative-strand RNA segments, which are thought to encode 10 viral proteins (PB2, PB1, PA, NP, NA, M1, M2, NS1, and NS2) (Lamb and Lai, 1980; Lamb et al., 1981). However, in the past few years, seven new proteins encoded by the virus genome have been identified, including PB1-F2 (Chen et al., 2001), PB1-N40 (Wise et al., 2009), PA-X (Jagger et al., 2012), M42 (Wise et al., 2012), NS3 (Selman et al., 2012), PA-N155, and PA-N182 (Muramoto et al., 2013). Among these novel discovered proteins, PA-X and PB1-F2 proteins have been discovered to have functions in regulating the host immune response and are suspected to play roles in the adaptation of influenza to mammalian hosts.

The PA-X protein, as a result of a +1 ribosomal frameshift, undergoes a change at a specific location in the PA mRNA that results in a change in activity, shares the same N-terminal 191 aa sequence with the corresponding PA protein, and has a unique C-terminal 41 aa (truncated) or 61 aa (full-length) sequence encoded by an overlapping open-reading frame (ORF) (Jagger et al., 2012). PA-X exhibits endonuclease activity though the same N-terminal amino acid sequence as the corresponding PA protein, and the C-terminal part of PA-X contributes to the suppression of host protein synthesis (Hayashi et al., 2015; Xu et al., 2016). Furthermore, PA-X can selectively degrade mRNAs transcripts produced by the host RNA polymerase II, but it ignores the activity of RNA polymerase I and III (Khaperskyy et al., 2016).

The multiple effects induced by the PA-X protein have been studied *in vitro* and *in vivo*. The PA-X protein can modulate the host immune response (Gao et al., 2015b) and has effects on viral replication and pathogenicity (Lee et al., 2017). In addition, phylogenetic analysis of over 3,000 PA gene sequences demonstrated that the truncated PA-X protein overwhelmingly comes from mammalian influenza, indicating that the PA-X protein may be associated with the adaptation of IAV from avian hosts to mammalian species (Shi et al., 2012).

The PB1-F2 protein is encoded by a +1 ORF in the PB1 sequence as a result of leaky ribosomal scanning, which is likely triggered by an optimal Kozak sequence at the start codon of PB1-F2 (Chen et al., 2001). Several functions of the PB1-F2 protein have been described, such as inducing cell death (Chen et al., 2001), host innate immune response regulation (Leymarie et al., 2013), and viral polymerase activity enhancement (Mazur et al., 2008). However, the effects caused by PB1-F2 in regard to virulence were cell type, strain, and host specific. Therefore, the PB1-F2 protein is known as a crucial virulence factor of IAV but the common mechanisms inducing the virulence change are still unclear. There are multiple versions of the PB1-F2 protein with different lengths, but generally avian-original PB1-F2 proteins consist of the full-length 90 aa and a truncated 11 aa version comes from human and swine influenza. Thus, several studies have suggested that truncated PB1-F2 proteins contribute to the adaptation of the virus to mammalian hosts (Alymova et al., 2014; Kamal et al., 2017).

Since the 2009 pandemic, when the H1N1 (Pdm09) influenza virus was discovered in North America and spread throughout the entire world, H1N1 has caused severe economic losses and remains a significant threat to public health. The Pdm09 viruses express both the truncated PB1-F2 (11 aa) and PA-X (232 aa) proteins, and the functions of these proteins have been well-characterized. Lee et al. (2017) found that the expression of PA-X increased viral virulence *in vitro* and *in vivo* when compared with a deficiency virus, Cal/09. For the PB1-F2 proteins, studies have found that restoring the truncated PB1-F2 protein of the Pdm09 virus has minimal effect on viral pathogenicity in mice and pigs (Hai et al., 2010; Pena et al., 2012). However, although some studies indicated the similarity of the PA-X and PB1-F2 proteins, such as in promoting mammalian adaptation and regulating the host immune response, the combined effects of the PB1-F2 and PA-X proteins on viral virulence as well as the host immune response are still unclear.

In this study, we used the 2009 pH1N1 A/Guangdong/1057/2010(GD1057) virus as a backbone and used a genetic reverse engineering system to generate four reconstructed viruses to investigate the comprehensive effects of the four types of expression forms of the PA-X and PB1-F2 proteins (truncated PA-X and PB1-F2, truncated PA-X with full-length PB1-F2, full-length PA-X with truncated PB1-F2, full-length PA-X and PB1-F2) on viral replication, pathogenicity and host innate response.

## Materials and Methods

### Ethics Statement

All animal studies were conducted under the Guide for the Care and Use of Laboratory Animals of the Ministry of Science and Technology of the People's Republic of China and were approved by the animal experimental ethics committee of the South China Agricultural University (approval number 2013-07).

### Cells and Viruses

Madin-Darby canine kidney (MDCK), human embryonic kidney cells (293T), and porcine kidney (PK-15) cells were cultured in Dulbecco's modified Eagle's medium (DMEM; Gibco, USA) complemented with 10% fetal bovine serum (FBS; Gibco, USA) and 1% penicillin-streptomycin (HyClone, USA) at 37°C and 5% CO_2_.

A/Guangdong/1057/2010 (GD1057) is a Pdm09 H1N1 influenza virus strain that was isolated in 2010 from human with severe respiratory symptoms in Guangdong, China (Table S1), and preserved in our laboratory. Virus stocks were propagated in MDCK cells for three passages and sequenced before use.

### Generation of Viruses by Reverse Genetic Engineering

All eight segments from GD1057 were amplified by RT-PCR (Table S2) and cloned into plasmid PHW2000 as described previously (Hoffmann et al., 2000), which were named WT_X. To rescue viruses expressing the full-length PA-X protein, a single codon mutation was introduced from UAG (stop) to UGG (tryptophan) at position 42 in the C-terminal domain of the PA-X protein based on WT_PA. The plasmid was named Pdm09_PAX_61. Three nucleotide substitutions (153A to C, 291A to G, and 381A to G) were introduced into the WT _PB1 plasmid to prolong the coding PB1-F2 protein from 11 to 90 aa (Table S3), and the resulting plasmid was named Pdm09_F2_90. None of the mutations in the plasmids changed PA or PB1 ORF production. Mutations were introduced with the Fast-directed Site-directed Mutagenesis kit (TIANGEN, China) by following the manufacturer's instructions, and every plasmid was confirmed by sequencing.

To analyze the effects of the PA-X and PB1-F2 proteins in GD1057, wild type (WT_PAX_41/F2_11) and three mutated recombinant viruses (Pdm09_PAX_41/F2_90, Pdm09_PAX_61/F2_11, Pdm09_PAX_61/F2_90) were rescued by the PHW2000 eight-plasmid system as described previously (Hoffmann et al., 2000). Briefly, a monolayer of 293T cells with ~90% confluence in six-well plates were cotransfected with eight constructed PHW2000 plasmids (WT_PB2, WT_PB1 or Pdm09_F2_90, WT_PA or Pdm09_PAX_61, WT_HA, WT_NP, WT_NA, WT_M, and WT_NS) encoding viral genomic RNA segments using Lipofectamine™ 3000 Transfection Reagent (Invitrogen). Then, 24 h of incubation, the supernatant of the 293T cell culture was harvested and passaged three times in MDCK cells to prepare the viral stocks. The rescued viruses were confirmed by virus genome sequencing.

### Viral Titration and Replication Kinetics

Before using the stock of viruses, TCID_50_ of each virus was inoculated in MDCK cells using a 10-fold serially diluted virus inoculated at 37° C and harvested after 48 h. The TCID_50_ value was calculated by the Reed and Muench method as previous studies (Xu et al., 2016; Lee et al., 2017). The experiments were done in triplicates. To evaluate the replication kinetics of the viruses, MDCK and PK-15 cells were infected with viruses at a multiplicity of infection (MOI) of 0.01. Supernatants of the infected cells were collected and stocked at 12, 24, 36, and 48 hpi. The virus titer was determined based on the TCID_50_ in the MDCK cells from the TCID_50_, and three independent repeat experiments were conducted to exclude any error.

### Viral RNP Polymerase Activity Assay

To evaluate the effects of the PA-X and PB1-F2 proteins on the polymerase activity, a ribonucleoprotein (RNP) mini-genome assay was conducted as described previously (Tan et al., 2014). Briefly, 293T cells in 12-well plates were cotransfected with 0.25 μg of each RNP complex expression plasmids (WT_PB2, WT_PB1 or Pdm09_F2_90, WT_PA or Pdm09_PAX_61, WT_NP), the luciferase reporter plasmid pPolI- Luci -NP and a 0.025 μg Renilla luciferase expressing plasmid as an internal control by using Lipofectamine 3000 (Invitrogen, USA), as recommended by the manufacturer. Cells transfected without the PA plasmid were used as a negative control. Then, 24 h post-transfection, cells in the wells were harvested, and luciferase signals were determined with a Dual-Luciferase Reporter Assay System (Promega, USA).

### Western Blot Analysis

Total cell protein lysates were extracted from the transfected 293T cells with CA630 lysis buffer (150 mM NaCl, 1% CA630 detergent, 50 mM Tris base [pH 8.0]). Proteins were separated by 12% sodium dodecyl sulfate-polyacrylamide gel electrophoresis (SDS-PAGE) and transferred to a polyvinylidene difluoride (PVDF) membrane (Merck Millipore, USA). Each PVDF membrane was blocked with 0.1% Tween 20 and 5% non-fat dry milk in Tris-buffered saline and subsequently incubated with a primary antibody. The primary antibodies were specific for influenza A virus PA (1:3,000, GeneTex, USA), influenza A virus PB1 (diluted 1:3,000, GeneTex, USA), influenza A virus NP (1:3,000, GeneTex, USA), and influenza A virus PA-X (diluted 1:2,000, polyclonal rabbit antiserum against the PA-X derived peptide (CAGLPTKVSHRTSPA) (diluted 1:3,000 Genscript, China). The secondary antibody used was either horseradish peroxidase (HRP)-conjugated anti-mouse antibody or HRP-conjugated anti-rabbit antibody (diluted 1:10,000 Beyotime USA) as appropriate. HRP presence was detected using a Western Lightning chemiluminescence kit (Amersham Pharmacia, Freiburg, Germany), following the manufacturer's protocol.

### GFP Expression Assay

293T cells in 6-wells plates were cotransfected with 400 ng of pEGFP-N1 plasmid (Clontech), 400 ng of PA (PAX_41 or PAX_61) plasmid and 400 ng of PB1 (PB1_F2_11 or PB1_F2_90) plasmid. 293T cells were cotransfected with 400 ng of pEGFP-N1, and 800 ng of empty PHW2000 was used for the control group. Then, 24 h post-transfection, the cells were harvested, and their mean fluorescent intensity was measured using flow cytometry (Beckman Coulter, USA) and FlowJo (treestar) software. The fluorescence intensity of cells was calculated after normalizing to the control group (taken as 100%). Three independent experiments were performed. Cell lysates from each group were used to evaluate the expression levels of PB1, PA, and GFP (1:1000, GeneTex, USA) by Western blotting. β-actin was used as a loading control. Detected protein bands were quantified using densitometry (Image-Pro Plus, Media Cybernetics) and the expression levels of PB1, PA, and GFP were normalized to β-actin.

### Mouse Infections

A total of 80 6-week-old female BALB/c mice (Guangdong Medical Laboratory Animal Center, China) were randomly divided into five groups (14 mice/group). Mice from each group were slightly anesthetized with dry ice and inoculated intranasally (IN) with 50 μL DMEM fluid containing 10^6^ TCID_50_ of each virus or 50 μL of virus-free DMEM. Mice were monitored daily for weight loss and clinical signs until 14 days post-inoculation. If a mouse had decreased in body weight of more than 25% compared with their original body weight, they were considered dead and were euthanized. At day 1, 3 and 5 days post-infection, three randomly selected mice in each group were euthanized, and their lungs were collected and stored in order to analyze virus titers, the host innate immune response and to conduct histopathology assays. To determine the virus titer in the lungs, 10% lung homogenates were made in DMEM with 1% antibiotic supplementation. Virus titers in the lung homogenates were determined by TCID_50_ assays on MDCK cells as mentioned above.

### Histopathology

At 5 days post-infection, lung tissues of euthanized mice were collected and immersed in 10% phosphate-buffered formalin then embedded in paraffin 48 h later. After that, samples from the same part of the lungs were cut into 5-μm-thick sections and one section from each sample was stained by hematoxylin and eosin (H&E).

### RT-qPCR

Total RNA in the lung homogenates was extracted by using TRIzol® Reagent RNA (Invitrogen) following the manufacturer's protocol. Total RNA from each sample was extracted and purified prior to cDNA synthesis completed by using a PrimeScriptTMII 1st Strand cDNA Synthesis Kit (Takara) with random primers. The mRNA expression level of cytokines/chemokines, including TNF-α, IL-6, NLRP3, caspase-1, IL-1β, and IL-18 (Table S4), were quantitated by real-time PCR assays (qRT-PCR). The qRT-PCR mixture for each sample contained 10 μL 2 × TB green Premix DimerEraser (Takara), 6 μL of RNase-free H_2_O, 1 μL of each primer and 2 μL of the cDNA template. The amplification program was as follows: 1 cycle of 95°C for 30 s; 40 cycles of 95°C for 5 s, 55°C for 30 s, and 72°C for 30 s; and finally, 1 cycle of 95°C for 5 s, 60°C for 1 min and 95°C for 5 s. Each cDNA template was tested with three experimental replicates to guarantee the quality of the data, and the results were calculated by the 2^−ΔΔ*Ct*^ method after normalization to the GAPHD gene expression level as an internal control.

### Statistical Analysis

All statistical analyses were performed using GraphPad Prism software version 5.00 (GraphPad Software Inc., San Diego, CA). Comparisons between two treatment means were conducted using two-tailed Student's *t*-test, whereas multiple comparisons were carried out by two-way analysis of variance (ANOVA) considering time and virus as factors. The differences were considered statistically significant at *P*-value < 0.05.

## Results

### Generation of Pdm09 H1N1 Viruses Expressing the Indicated Full-Length or Truncated PA-X and PB1-F2 Proteins

**Figure 1:**
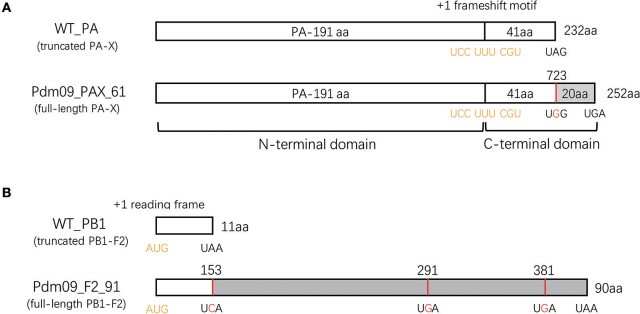
Generation of reassortment viruses expressing full-length or truncated PA-X and PB1-F2 proteins. **(A)** Schematic diagram of the PA sequence expressing the PA-X proteins. A conserved motif in the 592 bp of the N-terminal PA sequence may cause +1 ribosomal frameshifting (indicated in yellow) when expressing PA proteins. Consequently, this frameshift produces a PA-X protein that contains the same N-terminal 191 aa as the PA protein and a unique 41 or 61 aa C-terminal domain. To rescue mutated viruses expressing the full-length PA-X protein, a single mutation (UAG to UGG, indicated with red) was induced at the stop codon to prolong the C-terminal PA-X protein from 41 to 61 aa. **(B)** Schematic representation of the PB1 sequence expressing PB1-F2 proteins. The PB1-F2 protein was translated by a +1 alternate ORF (indicated in yellow) in the PB1 sequence. A virus encoding the full-length PB1-F2 protein was constructed by introducing three mutations (A153C, A 291G, and A 381G, highlighted with red) in the PB1 plasmid.

To evaluate the effects of the PA-X and PB1-F2 proteins on viral replicability, pathogenicity and host innate immune response, four viruses based on the GD1057 backbone were rescued after considering various combinations of truncated or full-length PA-X and PB1-F2 proteins. Wild type (WT_PAX_41/F2_11) virus expressed truncated 232 aa PA-X and 11 aa PB1-F2 proteins. The Pdm09_PAX_41/F2_90 virus was constructed by introducing three mutations into stop codons (153A to C, 291A to G and 381A to G) in the PB1 sequence, which consequently expressed truncated 232 aa PA-X and full-length 90 aa PB1-F2 proteins (Figure 1B). Pdm09_PAX_61/F2_11 was rescued after substituting the stop codon with tryptophan at the 42nd amino acid position of X-ORF in the PA sequence, and it expressed the full-length 252 aa PA-X protein and truncated 11 aa PB1-F2 protein (Figure 1A). Pdm09_PAX_61/F2_90 was rescued with the constructed plasmids and expressed full-length 252 aa PA-X and 90 aa PB1-F2 proteins. The PA-X protein could be detected in 293T cells from all four virus transfection groups (Figure S1), but we failed to detect PB1-F2 with a polyclonal antibody by western blotting.

### Full-Length PA-X and PB1-F2 Increase Virus Early Replication *in vitro* via Improving the Expression of Polymerase Components

**Figure 2:**
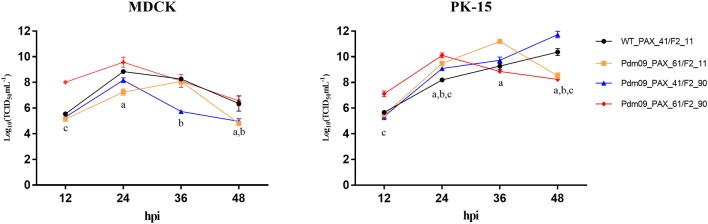
Viral growth kinetics of rescue viruses in MDCK and PK-15 cells. MDCK and PK-15 cells were inoculated with rescue viruses at a MOI of 0.01. The supernatants were harvested at the indicated time points until 48 hpi. Each point on the lines indicates the mean ± SD of three independent experiments and significant differences (*p* < 0.05) between infected groups are marked by a, b, and c (a: WT_PAX-41/F2-11 and Pdm09_PAX_61/F2_11; b: WT_PAX_41/F2_11 and Pdm09_PAX_41/F2_90; c: WT_PAX_41/F2_11 and Pdm09_PAX_61/F2_90).

To characterize all four reconstructed viruses *in vitro*, we inoculated each virus into MDCK and PK-15 cells with MOI 0.01 (Tables S5, S6). Both Pdm09_PAX_61/F2_11 and Pdm09_PAX_41/F2_90 viruses replicated slower in the MDCK cells and faster in the PK-15 cells at 24 and 36 hpi compared with the WT_PAX_41/F2_11. Interestingly, Pdm09_PAX_61/F2_90 grew to a high titer (*p* < 0.05) significantly faster than the other three viruses before 24 hpi in both cell lines (Tables S7, S8); however, the high titer of Pdm09_PAX_61/F2_90 could not be maintained after 24 hpi (Figure 2). The data demonstrated that the simultaneous expression of PA-X and PB1-F2 proteins enhanced virus replication in the early infection stage *in vitro*.

**Figure 3:**
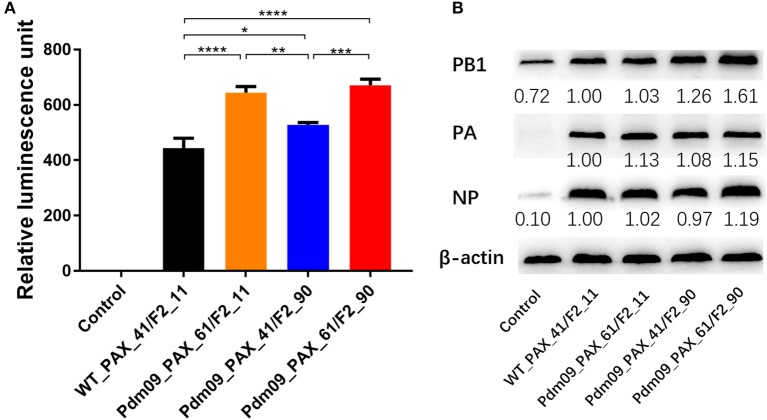
Effects of different expression of PA-X and PB1-F2 proteins on polymerase activity and expression of RNP components. **(A)** Comparison of polymerase activities of vRNPs with the indicated different expression of PA-X and PB1-F2 proteins in 293T cells. Relative luminescence units are presented as the mean ± SD from three independent experiments. **(B)** Expression of RNP components from 293T cells 24 h post-transfection with different combinations of individual RNP plasmids. The expression level of the RNP component relative to the expression of the control, β-actin, was determined by densitometry using BandScan software, version 5.0. Data are representative of the mean of three independent experiments and were standardized to those for WT_PAX_41/F2_11 (taken as 100%).

To measure whether the changes induced by the PA-X and PB1-F2 proteins were mediated via impacts on the polymerase activity, a dual-luciferase reporter assay was conducted to evaluate the viral polymerase activity. The results indicated that RNPs from WT_PAX_41/F2_11 and Pdm09_PAX_41/F2_90 showed significantly lower (*p* < 0.05) polymerase activity when compared with RNPs from Pdm09_PAX_61/F2_11 and Pdm09_PAX_61/F2_90 (Figure 3). This decreased activity indicated that the expression of the full-length PA-X protein could increase the polymerase activity compared with the truncated PA-X. Furthermore, plasmids encoding the full-length PB1-F2 protein slightly enhanced the polymerase activity when compared with the truncated one. Moreover, the polymerase complex from Pdm09_PAX_61/F2_90 harbored the strongest polymerase activity (Figure 3).

To further investigate the effects of different expression of PA-X and PB1-F2 proteins on viral RNA polymerase activity, western blot assays were conducted to determine the expression level of RNP component proteins in 293T cells 24 h post-transfection. Our results showed that viruses with the full-length PA-X protein resulted in a higher (*p* < 0.0001) PA expression level compared with viruses with the truncated PA-X. Furthermore, viruses with the full-length PB1-F2 protein could accumulate more PB1 protein (*p* < 0.001) than the truncated PB1-F2 version. In addition, viruses simultaneously expressing full-length PA-X and PB1-F2 could accumulate PB1 and NP proteins significantly faster (*p* < 0.0001) than other three groups of viruses (Figure 3). In summary, simultaneous expression of full-length PA-X and PB1-F2 proteins in GD1057 could enhance viral replication in the early infection period; expression of the full-length proteins only slightly increased viral RNA polymerase activity but significantly increased the expression level of viral RNP components.

### Different Forms of the PB1-F2 Protein May Induce Effects on the Host Shut-Off Activity of the PA-X Protein

**Figure 4:**
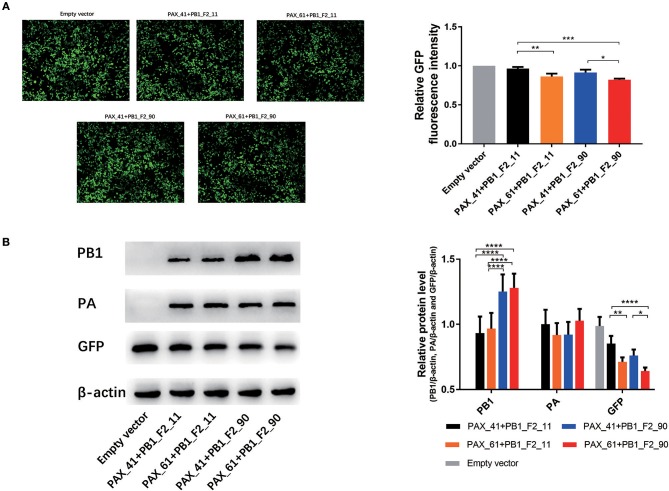
Effects of different expression of PA-X and PB1-F2 proteins on cotransfected GFP expression. 293T cells were cotransfected with the GFP expression plasmid and the indicated PA and PB1 expression plasmids encoding different PA-X and PB1-F2 forms. **(A)** Fluorescence images of GFP expression were captured 24 h post-transfection with the indicated PA and PB1 expression plasmids. The control group was cotransfected with the GFP expression vector along with empty vectors. Relative GFP fluorescence intensity was determined with flow cytometry and data were presented after normalization against the control group (taken as 100%). Three independent experiments were conducted. **(B)**. Expressed PB1, PA, and GFP protein were determined by Western blot analysis. β-actin protein was used as a loading control. The protein bands were quantified by densitometry. Relative protein levels of PB1, PA, and GFP as compared with β-actin are shown as histograms. Differences between each group were considered statistically significant at *p*-values of <0.05. The results shown are representative data from three independent experiments. The significant differences between the groups were marked with asterisks (^*^p < 0.05, ^**^p < 0.01, ^***^p < 0.001, ^****^p < 0.0001).

Several studies have revealed that PA-X protein contributes to the global host shut-off activity of influenza virus. To measure whether PB1-F2 proteins have impacts on the shutoff activity of PA-X protein, 293T cells were cotransfected with plasmids expressing GFP, individual plasmids expressing PA (expressing full-length or truncated PA-X) and corresponding plasmids expressing PB1 (expressing full-length or truncated PB1-F2) for 24 h. Relative GFP expression was determined through the assessment of fluorescent intensity (Figure S2) and western blot assays. GFP expression of PAX_61+PB1_F2_90 was significant lower (*p* < 0.05) than PAX_41+PB1_F2_11 and PAX_41+PB1_F2_90. PAX_61+PB1_F2_11 values were significantly lower *(p* < 0.05) than PAX_41+PB1_F2_11. Furthermore, a modest decrease in GFP expression was observed after replacing truncated PB1-F2 with full-length protein (Figure 4). These data suggest that the expression of full-length or truncated PB1-F2 may influence the shutoff activity of PA-X in GD1057 viruses.

### Full-Length PB1-F2 and PA-X Proteins Enhance Viral Pathogenicity During the Early Infection Stage in Mice

**Figure 5:**
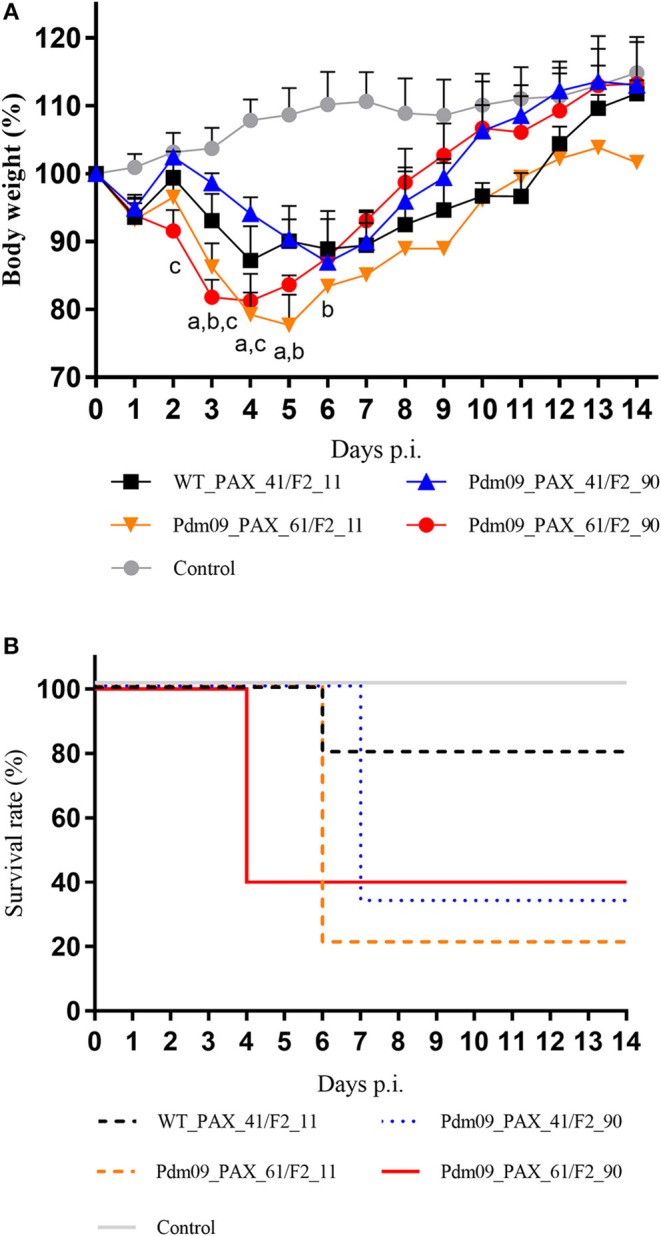
Virulence of different reconstructed viruses in BALB/c mice. **(A)** The body weight of the mice was monitored and recorded until 14 dpi and the results are presented as percentage of the weight on the day of inoculation (day 0). Points of each group show the means and error bars (SD) and significant differences (*p* < 0.05) between infected groups are marked by a, b, and c (a: WT_PAX_41/F2_11 and Pdm09_PAX_61/F2_11; b: WT_PAX_41/F2_11 and Pdm09_PAX_41/F2_90; c: WT_PAX_41/F2_11 and Pdm09_PAX_61/F2_90). **(B)** Survival rates of each experimental group are presented in percentages.

To investigate the effects of different forms of PA-X and PB1-F2 proteins on the virulence of the Pdm09 virus *in vivo*, mice from each group were intranasally inoculated with 10^6^ TCID_50_ of the corresponding viruses and monitored daily for clinical signs and weight loss. Classic clinical signs of influenza could be observed in all infected mice from the experimental groups, such as depression and reduced activity. Furthermore, weight loss could be measured at 3 dpi when compared with the control animals. It is obvious that viruses (Pdm09_PAX_61/F2_11 and Pdm09_PAX_61/F2_90) expressing full-length PA-X protein induced more body weight loss (79.23 and 81.25% by 4 dpi, respectively) than viruses expressing truncated proteins (WT_PAX_41/F2_11, 87.22% by 4 dpi and Pdm09_PAX_41/F2_90, 86.92% by 6 dpi). It was notable the viruses expressing full-length PB1-F2 and PA-X showed more virulence compared with other three viruses from the 1st to the 4th dpi (Figure 5A). To the survival rate, Pdm09_PAX_61/F2_90 exhibited 60% mortality on 4 dpi, which was much higher compared with the other three viruses (Figure 5B).

**Figure 6:**
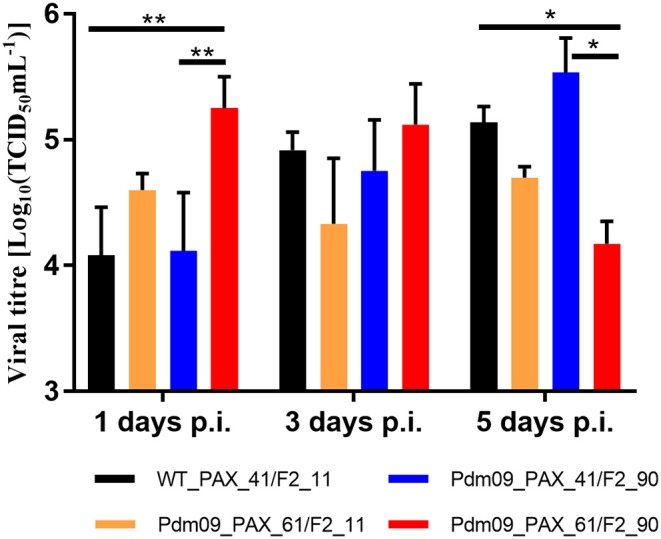
Virus titers of rescue viruses in mouse lungs were determined at 1, 3, and 5 dpi by TCID_50_ in MDCK cells. Values are the mean ± SD of the results from three mice. The significant differences between groups are marked with asterisks (^*^p < 0.05, ^**^p < 0.01).

Virus titers in the lungs of the infected mice were measured by TCID_50_, and the results (Table S9) demonstrated that Pdm09_PAX_61/F2_90 grew to a significantly higher titer 1 dpi but reached a lower titer 5 dpi when compared with the WT_PAX_41/F2_11 and Pdm09_PAX_61/F2_11 viruses (Figure 6).

**Figure 7:**
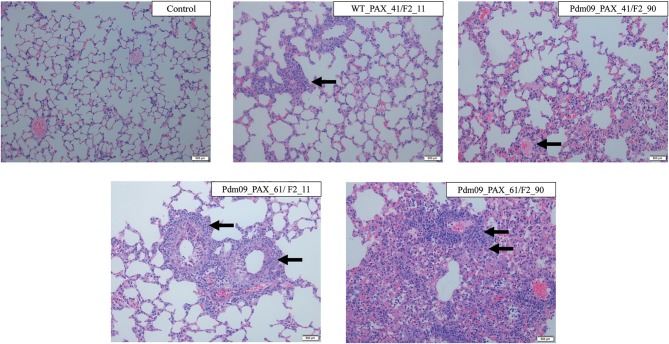
Histopathological changes of mouse lungs infected with the indicated viruses at 5 dpi. Negative control group: No lesions in the bronchiole could be observed. WT_PAX_41/F2_11 and Pdm09_41/F2_90: A small number of neutrophils were present in the bronchiole lumen and there was mild inflammatory consolidation. Pdm09_61/F2_11: The bronchiole was filled with neutrophils and there was moderate mild inflammatory consolidation. Pdm09_61/F2_90: The bronchiole was filled with neutrophils and abundant inflammatory consolidation could be observed. The infiltration of neutrophils was marked by arrows.

The pathogenicity induced by the reconstructed viruses was further demonstrated by histopathology analysis of the lung tissues collected at 5 dpi. Typical influenza pneumonia could be observed in all four infection groups. Viruses expressing full-length PA-X protein induced more lesions than viruses with a truncated protein and produced more cellular infiltration and inflammatory consolidation, indicated that the full-length PA-X protein increased viral virulence in mice compared with the truncated version (Figure 7). Moreover, the Pdm09_PAX_61/F2_90 virus induced the most severe lesions among four viruses; the bronchioles were saturated with neutrophils, and the adjacent alveolar lumina were obviously expanded by lymphocytes and histocytes, indicating a longer duration of lung inflammation. These results suggested that simultaneous expression of full-length PB1-F2 and PA-X proteins enhanced the pathogenicity of the GD1057 virus in the early infection stage in mice.

### PB1-F2 and PA-X Proteins Simultaneously Regulate the Inflammatory Response During the Early Infection Stage in Mice

**Figure 8:**
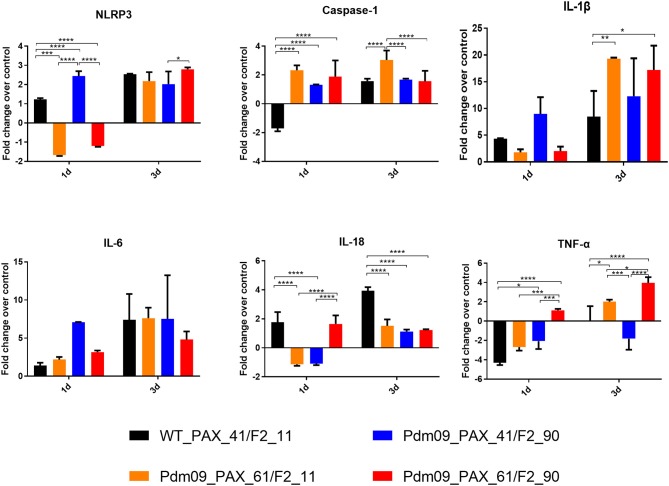
The expression levels of pro-inflammatory cytokines mRNA in the lungs of the mice infected with the reconstructed viruses. The mRNA expression levels of the indicated mouse cytokines were quantified by qRT-PCR. The expression fold change of each group was calculated relative to the control group after normalization to the internal control GAPDH gene using the 2-ΔΔCt method. Data represent the mean ± SD of three mice in each group on the days indicated. The asterisks (^*^) indicate a statistically significant difference between groups (^*^p < 0.05. ^**^p < 0.01, ^***^p < 0.001, ^****^p< 0.0001).

To further estimate the immune response caused by different expression patterns of the PA-X and PB1-F2 proteins, cytokine genes related to the inflammatory response in the lungs of infected mice were determined by qPCR. For the PA-X proteins, the Pdm09_PAX_61/F2_11 virus suppressed the expression levels of NLRP3, IL-18, and IL-1B at 1 dpi and increased the expression levels of caspase-1, IL-1B and TNF-a at 3 dpi when compared with WT_PAX_41/F2_11. For the PB1-F2 proteins, the Pdm09_PAX_41/F2_90 virus increased the levels of NLRP3, caspase-1, IL-1B, and IL-6 at 1 dpi and decreased the levels of IL-18 and TNF-a at later time points (3 dpi). Interestingly, the Pdm09_PAX_61/F2_90 virus upregulated the expression levels of IL-18 and TNF-a at 1 dpi when compared with Pdm09_PAX_61/F2_11. Furthermore, the Pdm09_PAX_61/F2_90 virus expressed higher levels of IL-18 and TNF-a at 1 dpi and higher levels of NLRP3 and TNF-a at 3 dpi when compared with the PAX_41/F2_90 virus (Figure 8). These data indicated that single expression of the truncated PA-X protein could suppress the early cytokine response, and single expression of PB1-F2 resulted in an increased early inflammatory response in the lungs of infected mice when compared with the wild type virus. Interestingly, following simultaneous expression of full-length PA-X and PB1-F2 proteins, some of the upregulated gene (NLRP3, IL-1B, and IL-6) expression induced by the full-length PB1-F2 was suppressed by the full-length PA-X. However, the full-length PB1-F2 protein could neutralize the suppression effect caused by the full-length PA-X protein and enhanced the expression level of some cytokine response genes, such as IL-8 and TNF-a. These results indicated that the PA-X and PB1-F2 proteins were simultaneously regulating the host immune response.

## Discussion

Since the PA-X protein was discovered in 2012, multiple characteristics of this protein have been described in detail, including its effects on viral pathogenicity and modulation of the host immune response and the host shutoff activity that suppresses host protein synthesis (Gao et al., 2015b; Hu et al., 2018). Another well-studied virulence factor is the PB1-F2 protein, which has been demonstrated to regulate antiviral innate immunity, enhance viral polymerase activity, induce cell death and alter virus virulence *in vivo* (Kamal et al., 2017). Functions of the PA-X and PB1-F2 proteins in the 2009 pH1N1 virus were individually studied by several researchers (Hai et al., 2010; Pena et al., 2012; Gao et al., 2015a; Lee et al., 2017). However, the combined effects of these two proteins are still unclear. In contrast to previous studies, we investigated the combined effects caused by different forms of the PA-X (232 or 252 aa) and PB1-F2 proteins (11 or 90 aa) in the 2009 pH1N1 virus in regard to viral replication, pathogenicity and host immune response.

A previous study has reported that the full-length PA-X protein from the Cal/09 virus leads to more efficient replication in cells, more viral polymerase activity and greater pathogenicity in mice compared with the truncated version (Lee et al., 2017). For the PB1-F2 protein, it has been indicated that the single restored truncated PB1-F2 protein of Cal/09 only has minimal effects on the replication efficiency and pathogenicity in mice and swine (Pena et al., 2012). Our *in vitro* results of single restored PA-X or PB1-F2 proteins were consistent with previous findings. When we simultaneously expressed both the full-length PA-X and PB1-F2 proteins in the 2009 pH1N1 virus, there was a significant increase of viral growth efficiency in both MDCK and PK-15 cells in the early infection stage when compared with the other three viral groups. Furthermore, a virus with full-length PA-X and PB1-F2 proteins only slightly increased the polymerase activity but significantly improved the viral RNP expression level when compared with the virus expressing full-length PA-X and truncated PB1-F2. These results indicated that the higher early viral replication induced by the presence of full-length PA-X and PB1-F2 proteins was mainly due to increased expression of the viral RNP components.

Our *in vivo* experiments exhibited consistent results with the *in vitro* experiments in that virus expression of the full-length PA-X and PB1-F2 proteins exhibited greater pathogenicity (weight loss, mortality and pathology) than the other three viruses in the early infection stage of mice. Our results indicated that the expression of full-length PA-X and PB1-F2 proteins enhanced viral replication *in vitro* and pathogenicity *in vivo* during the early infection stage.

Both PA-X and PB1-F2 proteins could regulate the host immune response. The expression of PA-X protein could suppress the inflammatory reactions. Lee et al. (2017) proved that the full-length PA-X suppressed the inflammatory response compared with the truncated one in Cal/09 virus infection. PB1-F2 proteins have been shown to interfere with the immune response through direct interaction with mitochondrial antiviral signaling (MAVS) protein and other components of the MAVS system (Le Goffic et al., 2011; Varga et al., 2012; Yoshizumi et al., 2014), resulting in an early inflammatory response (Pena et al., 2012). In the present study, our results were consistent with previous research in that single restored PA-X could suppress cytokine and chemokine responses in early infection and expression of full-length PB1-F2 by itself could increase the expression level of genes related to cytokines and chemokines.

Interestingly, after comparing data from viruses that contain full-length PA-X and PB1-F2 with data from the other three reconstructed viruses, our results indicated that the early inflammatory response caused by full-length PB1-F2 could be suppressed by full-length PA-X. However, simultaneous expression of PA-X and PB1-F2 enhanced the expression level of IL-18 and TNF-a during the early infection period, although individual expression of full-length PA-X or PB1-F2 could decrease the expression level of these two genes. These results indicated that the PA-X and PB1-F2 proteins could simultaneously regulate the host immune response. Detailed mechanism of the interplay between the PA-X and the PB1-F2 proteins warrants further investigation.

Influenza virus infection leads to the suppression of global host protein synthesis in infected cells, also known as host shutoff activity (Levene and Gaglia, 2018). The influenza host shutoff activity results in a decrease in the host innate immune response and alters ribosome activity toward translation of viral mRNAs. After Jagger et al. (2012) first demonstrated that PA-X can suppress the gene expression of cotransfected plasmids, several studies have showed that PA-X makes a contribution to influenza host shutoff activity in different viral subtypes in multiple animal models (Gao et al., 2015b). Gao et al. (2015b) further demonstrated that the additional 20 aa from the C-terminal end of PA-X protein has a strong shutoff activity. In contrast to the PA and PA-X proteins, there has been no report about PB1 and PB1-F2 proteins being involved in host shutoff effects. Our results showed that the expression of full-length PA-X increased the host shutoff activity as previous research (Gao et al., 2015b). Of note, prolonging the truncated PB1-F2 to full-length form may slightly enhance the host shutoff activity induced by PA-X in the GD1057 virus. Because of PB1-F2 host and strain specific patterns, further research is needed to unveil the mechanism between PA-X and PB1-F2.

In summary, our research indicated that the simultaneous expression of full-length PA-X and PB1-F2 proteins in the 2009 pH1N1 virus increased viral replication during the early infection stage *in vitro*, likely via promoting the expression of the viral RNP complex protein, and enhancing viral virulence *in vivo* mainly through simultaneously mediated host immune response. Moreover, our research indicated that different forms of PB1-F2 may have impacts on the host shutoff activity of PA-X protein in GD1057. Our study provides a better understanding of the functions of different forms of PA-X and PB1-F2 proteins in regard to viral pathogenicity and host immune response.

## Data Availability

The datasets generated for this study are available on request to the corresponding author.

## Author Contributions

JM, ShuL, KL, and XW conducted the experiments. JM analyzed the data and wrote the paper. ShoL designed the experiments and revised the paper.

### Conflict of Interest Statement

The authors declare that the research was conducted in the absence of any commercial or financial relationships that could be construed as a potential conflict of interest.
