# Supplementary material for: Effects of the PA-X and PB1-F2 Proteins on the Virulence of the 2009 Pandemic H1N1 Influenza A Virus in Mice
Source: Front Cell Infect Microbiol. 2019 Sep 3;9:315. doi: 10.3389/fcimb.2019.00315 (PMC6734165; doi:10.3389/fcimb.2019.00315)

Table S1: Genbank number of eight segments of A/Guangdong/1057/2010 (GD1057).

| Sequence | Genbank number |
| --- | --- |
| PB2 | MN244305 |
| PB1 | MN244306 |
| PA | MN244307 |
| HA | MN244309 |
| NP | MN244308 |
| NA | MN244310 |
| M | MN244311 |
| NS1 | MN244312 |

Table S2: Primers for RT-PCR.

| Primers | Sequence |
| --- | --- |
| CU | AGCAAAAGCAGG |
| HA-BSAI-F | TATTGGTCTCAGGGAGCAAAAGCAGGGG |
| HA-BSAI-R | ATATGGTCTCGTATTAGTAGAAACAAGGGTGTTTT |
| NA-BSMBI-F | TATTCGTCTCAGGGAGCAAAAGCAGGAGTAA |
| NA-BSMBI-R | ATATCGTCTCGTATTAGTAGAAACAAGGAGTTTTT |
| NP-AarI-F | ATCACCTGCTGGCGGGAGCAAAAGCAGGGTA |
| NP-AarI-R | TATCACCTGCGCCGTATTAGTAGAAACAAGGGTATTTT |
| M-BSMBI-F | TATTCGTCTCAGGGAGCAAAAGCAGGTAGATATTG |
| M-BSMBI-R | ATATCGTCTCGTATTAGTAGAAACAAGGTAGTTTTTAC |
| NS-BSMBI-F | TATTCGTCTCAGGGAGCAAAAGCAGGGTG |
| NS-BSMBI-R | ATATCGTCTCGTATTAGTAGAAACAAGGGTGTTTT |
| PA-AarI-F | ATCACCTGCTGGCGGGAGCGAAAGCAGGTAC |
| PA-AarI-R | TATCACCTGCGCCGTATTAGTAGAAACAAGGTACT |
| PB1-AarI-F | ATCACCTGCTGGCGGGAGCGAAAGCAGGCA |
| PB1-AarI-R | TATCACCTGCGCCGTATTAGTAGAAACAAGGCATTT |
| PB2-BbsI-F | TATTGAAGACTAGGGAGCGAAAGCAGGTC |
| PB2-BbsI-R | ATATGAAGACTGTATTAGTAGAAACAAGGTCGTTT |
| pHW2000-F | CTAGAGAACCCACTGCTTAC |
| pHW2000-R | TTGGGGACAGGTGTCCGTGT |
| pJET 1.2F | CGACTCACTATAGGGAGAGCGGC |
| pJET 1.2R | AAGAACATCGATTTTCCATGGCAG |

Table S3: Primers for mutation.

| Primers | Sequence |
| --- | --- |
| H1N1-PA-X-61aa-F | TTAGAGCCTATGTGGATGGATTCGAGC |
| H1N1-PA-X-61aa-R | CCACATAGGCTCTAAAGTTTTCAAGGCT |
| H1N1-PB1-F2-F A153C | CCATGGACACAGTCAACAGAACACACC |
| H1N1-PB1-F2-R A153C | TGACTGTGTCCATGGTGTATCCTGTTCC |
| H1N1-PB1-F2-F A291G | CAGACTGTGTTCTGGAGGCTATGGC |
| H1N1-PB1-F2-R A291G | CCAGAACACAGTCTGTTTGTGCATA |
| H1N1-PB1-F2-F A381G | CAAACAAGGGTGGATAAACTAACTC |
| H1N1-PB1-F2-R A381G | AGTTTATCCACCCTTGTTTGTTGAA |

Table S4: Primers for Q-PCR.

| Primers | Sequence |
| --- | --- |
| GAPDH-F | TGGCCTTCCGTGTTCCTAC |
| GAPDH-R | GAGTTGCTGTTGAAGTCGCA |
| NLRP3-F | ATTACCCGCCCGAGAAAGG |
| NLRP3-R | TCGCAGCAAAGATCCACACAG |
| Caspase-1-F | ACAAGGCACGGGACCTATG |
| Caspase-1-R | TCCCAGTCAGTCCTGGAAATG |
| IL-1β-F | GCAACTGTTCCTGAACTCAACT |
| IL-1β-R | ATCTTTTGGGGTCCGTCAACT |
| IL-6-F | TAGTCCTTCCTACCCCAATTTCC |
| IL-6-R | TTGGTCCTTAGCCACTCCTTC |
| IL-18-F | GACTCTTGCGTCAACTTCAAGG |
| IL-18-R | CAGGCTGTCTTTTGTCAACGA |
| TNF-α-F | CCTGTAGCCCACGTCGTAG |
| TNF-α-R | GGGAGTAGACAAGGTACAACCC |

Table S5: Log_10_(TCID_50_mL^-1^) of stock viruses before inoculating in MDCK and PK-15.

| Pdm09_PAX_41/F2_11 | Pdm09_PAX_61/F2_11 | Pdm09_PAX_41/F2_90 | Pdm09_PAX_61/F2_90 |
| --- | --- | --- | --- |
| 8.5 | 8.39 | 8.61 | 7.71 |
| 8.32 | 8.34 | 8.69 | 7.52 |
| 8.61 | 8.44 | 8.5 | 7.9 |

Table S6: Log_10_(TCID_50_mL^-1^) of stock viruses before inoculating in mice.

| Pdm09_PAX_41/F2_11 | Pdm09_PAX_61/F2_11 | Pdm09_PAX_41/F2_90 | Pdm09_PAX_61/F2_90 |
| --- | --- | --- | --- |
| 8.2 | 8.75 | 8.5 | 8.33 |
| 8.2 | 8.58 | 8.43 | 8.24 |
| 8.2 | 8.86 | 8.6 | 8.39 |

| dpi | Pdm09_PAX_41/F2_11 | Pdm09_PAX_61/F2_11 | Pdm09_PAX_41/F2_90 | Pdm09_PAX_61/F2_90 |
| --- | --- | --- | --- | --- |
| 12hpi | 5.64 | 5.6 | 5.64 | 7.36 |
|  | 5.84 | 5.47 | 5.16 | 6.88 |
|  | 5.5 | 5.33 | 5.25 | 7.16 |
| 24hpi | 8 | 9.5 | 9 | 10 |
|  | 8.33 | 9.5 | 9 | 10.33 |
|  | 8.25 | 9.45 | 9.25 | 10 |
| 36hpi | 9 | 11.33 | 9.5 | 8.67 |
|  | 9.5 | 11 | 10 | 9 |
|  | 9.33 | 11.25 | 9.67 | 8.88 |
| 48hpi | 10.67 | 8.67 | 11.5 | 8.23 |
|  | 10.16 | 8.63 | 12 | 8.25 |
|  | 10.25 | 8.25 | 11.68 | 8.25 |

Table S7: Log_10_(TCID_50_mL^-1^) in MDCK cells.

Table S8: Log_10_(TCID_50_mL^-1^) in PK-15 cells.

| hpi | Pdm09_PAX_41/F2_11 | Pdm09_PAX_61/F2_11 | Pdm09_PAX_41/F2_90 | Pdm09_PAX_61/F2_90 |
| --- | --- | --- | --- | --- |
| 12hpi | 5.69 | 5.16 | 5.31 | 8.16 |
|  | 5.5 | 5 | 5.31 | 7.88 |
|  | 5.45 | 5.25 | 5.25 | 8 |
| 24hpi | 9 | 7.25 | 8.16 | 10 |
|  | 8.83 | 7 | 8 | 9.23 |
|  | 8.71 | 7.5 | 8.36 | 9.5 |
| 36hpi | 8.39 | 8.23 | 5.63 | 7.67 |
|  | 8.23 | 7.83 | 5.83 | 8.67 |
|  | 8.17 | 8.16 | 5.75 | 8 |
| 48hpi | 7 | 5 | 4.75 | 6.23 |
|  | 5.83 | 4.67 | 5 | 7 |
|  | 6.25 | 4.75 | 5.15 | 5.45 |

Table S9: Log_10_(TCID_50_mL^-1^) in lungs of infected mice.

| dpi | Pdm09_PAX_41/F2_11 | Pdm09_PAX_61/F2_11 | Pdm09_PAX_41/F2_90 | Pdm09_PAX_61/F2_90 |
| --- | --- | --- | --- | --- |
| 1dpi | 3.75 | 4.55 | 3.6 | 5.5 |
|  | 4.5 | 4.5 | 4.5 | 5 |
|  | 4 | 4.75 | 4.25 | 5.25 |
| 3dpi | 5 | 3.75 | 4.5 | 5.25 |
|  | 4.75 | 4.75 | 5.16 | 5.36 |
|  | 5 | 4.5 | 4.75 | 4.75 |
| 5dpi | 5.16 | 4.6 | 5.84 | 4.16 |
|  | 5 | 4.75 | 5.31 | 4 |
|  | 5.25 | 4.75 | 5.45 | 4.36 |

Figure S1: PA-X detected by western blotting with specific antibody.


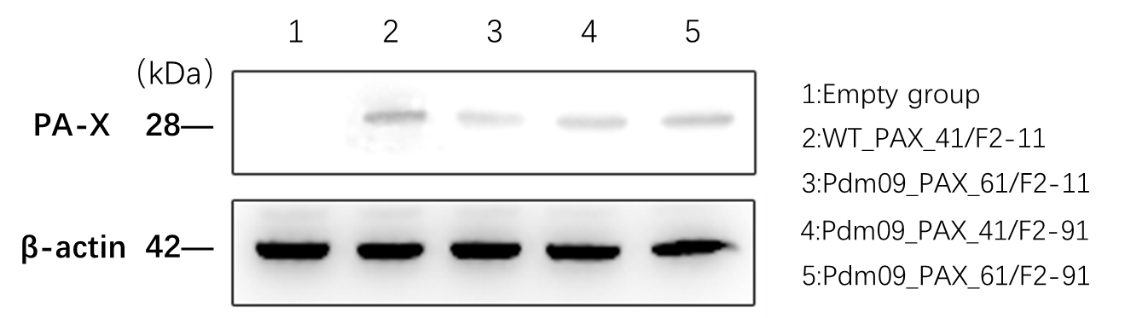


Figure S2: The mean fluorescent intensity was calculated using FlowJo (TreeStar) software.


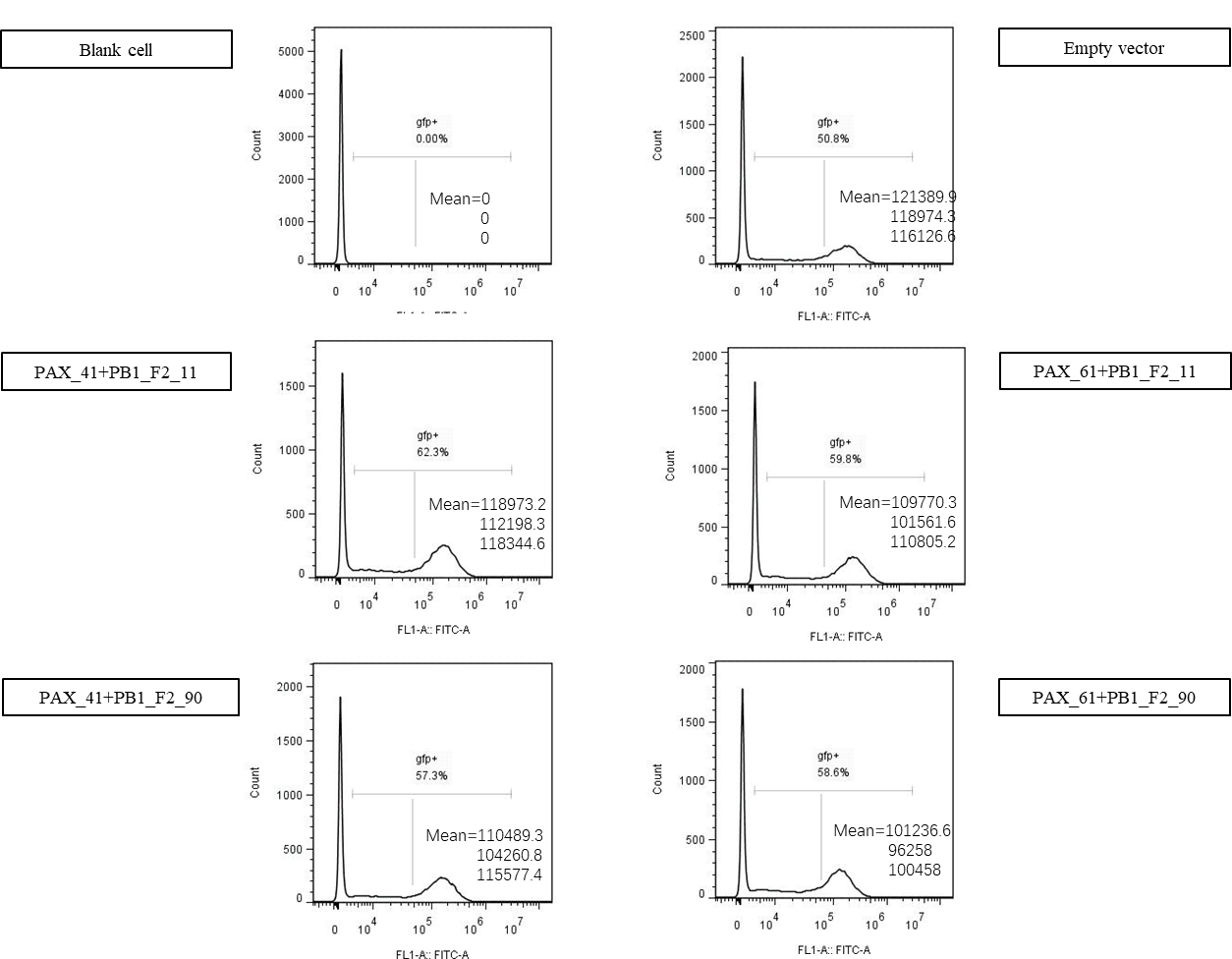

Supplement: Supplementary file 1 [file Data_Sheet_1.docx]
